# Premenstrual dysphoric disorder—an undervalued diagnosis? Preliminary results of a prospective study on Hungarian women

**DOI:** 10.1192/j.eurpsy.2024.287

**Published:** 2024-08-27

**Authors:** B. Pataki, B. L. Kiss, I. Juhász, S. Kálmán, I. Kovács

**Affiliations:** ^1^Department of Psychiatry, Albert Szent-Györgyi Medical School, University of Szeged; ^2^Albert Szent-Györgyi Medical School, University of Szeged, Szeged, Hungary

## Abstract

**Introduction:**

The premenstrual dysphoric disorder (PMDD) is a new distinct diagnostic entity in the Fifth Edition of the Diagnostic and Statistical Manual of Mental Disorders (DSM-5). However, the severe premenstrual (PM) symptoms associated with PMDD result in functional impairment, globally, it remains highly underdiagnosed, underscoring the need for enhanced clinical recognition.

**Objectives:**

This ongoing study aims to assess the prevalence and symptom profile of PMDD in a sample of Hungarian women. It is part of a comprehensive research process aiming to validate a prospective PMDD diagnostic questionnaire (Daily Record of Severity of Problems, DRSP) in order to facilitate the diagnosis of the disorder.

**Methods:**

The study was performed in three steps. Firstly, retrospective data were collected from 112 women. Probable PMDD was assessed using the DSM-5 Based Screening Tool, while anxio-depressive symptoms and well-being were evaluated using the Beck Depression Inventory, the state subscale of the State-Trait Anxiety Inventory, and the WHO Well-Being Scale. Subsequently, prospective data were obtained from 9 women who completed the DRSP along with the aforementioned mood questionnaires during both their PM and follicular phases.

**Results:**

In the first research phase, the sample was divided into women with probable PMDD diagnosis (PMDD group, n=68) and women without probable PMDD diagnosis (nonPMDD group, n=45) based on the DSM-5-Based Screening Tool. The PMDD group reported significantly more severe depressive (F(1; 56.2) = 19.394, *p*≤0.001) and anxiety (F(1; 35.6)=17.714, *p*≤0.001) symptoms and lower well-being (F(1; 44.3)=4.288, *p*=0.04) compared to the non-PMDD group, irrespective of the menstrual phase they experienced.

In the second and third research phases based on the DRSP, the sample was divided into women with probable PMDD diagnosis (PMDD group, n= 3) and those without probable PMDD diagnosis (nonPMDD group, n=6). A statistically significant association was observed between the classifications according to the DSM-5 Based Screening Tool and the DRSP (*p*=0.048; Cramer’s V=0.79). The PMDD group showed a tendency of lower well-being and more severe anxio-depressive symptoms than the nonPMDD group (Well-being: between phases *p*=0.93, between groups *p*=0.06; BDI-II: between phases *p*=0.79, between groups *p*=0.07; STAI-S: between phases *p*=0.87, between groups *p*=0.17).

**Conclusions:**

The prevalence of PMDD was high in our sample. Women with probable PMDD retrospectively reported substantial affective difficulties and a decline in subjective well-being, regardless of their menstrual cycle. Prospective preliminary findings suggest a trend toward differentiation associated with probable PMDD. These results highlight the need for prospective clinical studies addressing the psychological symptoms of women with PM issues and the importance of appropriate treatment of the clinical appearance of PMDD.

**Disclosure of Interest:**

None Declared

